# The Role of Coagulase-Negative Staphylococci Biofilms on Late-Onset Sepsis: Current Challenges and Emerging Diagnostics and Therapies

**DOI:** 10.3390/antibiotics12030554

**Published:** 2023-03-10

**Authors:** Angela França

**Affiliations:** 1 Centre of Biological Engineering, LIBRO—Laboratório de Investigação em Biofilmes Rosário Oliveira, University of Minho, Campus de Gualtar, 4710-057 Braga, Portugal; afranca@ceb.uminho.pt; 2 LABBELS—Associate Laboratory in Biotechnology and Bioengineering and Microelectromechanical Systems, Braga and Guimarães, Portugal

**Keywords:** neonatal sepsis, late-onset sepsis, coagulase-negative staphylococci, diagnosis, treatment, antibiotic resistance, antibiotic tolerance, biofilms

## Abstract

Infections are one of the most significant complications of neonates, especially those born preterm, with sepsis as one of the principal causes of mortality. Coagulase-negative staphylococci (CoNS), a group of staphylococcal species that naturally inhabit healthy human skin and mucosa, are the most common cause of late-onset sepsis, especially in preterms. One of the risk factors for the development of CoNS infections is the presence of implanted biomedical devices, which are frequently used for medications and/or nutrient delivery, as they serve as a scaffold for biofilm formation. The major concerns related to CoNS infections have to do with the increasing resistance to multiple antibiotics observed among this bacterial group and biofilm cells’ increased tolerance to antibiotics. As such, the treatment of CoNS biofilm-associated infections with antibiotics is increasingly challenging and considering that antibiotics remain the primary form of treatment, this issue will likely persist in upcoming years. For that reason, the development of innovative and efficient therapeutic measures is of utmost importance. This narrative review assesses the current challenges and emerging diagnostic tools and therapies for the treatment of CoNS biofilm-associated infections, with a special focus on late-onset sepsis.

## 1. Introduction

According to the World Health Organization, 15 million babies are born preterm (before completing 37 weeks of gestation) every year [1], with preterm birth complications responsible for 1 million deaths annually [2]. One of the most significant complications in preterm infants is the development of infections, with neonatal sepsis (NS) as one of the principal causes of mortality and long-term neurological impairments [2,3,4]. The Global Burden of Disease estimated, in 2017, that there were 1.3 million annual incident cases of NS worldwide [5], resulting in 203,000 attributable deaths [6].

The definition of sepsis in adults was recently redefined, by the Third International Consensus Definitions for Sepsis and Septic Shock (Sepsis-3) taskforce, as a life-threatening and dysregulated host response to an infection [7]. However, when it comes to sepsis in newborns, there is no international consensus, with criteria used to define NS varying among studies [8]. As such, an international consensus is necessary to standardize the criteria for NS definition, which is essential for accurate diagnoses and positive outcomes [8]. In general, NS is defined as a clinical syndrome detected during the first 28 days of life, which involves systemic signs of infection, circulatory shock and multisystem organ failure [9]. NS can be further classified as either early- or late-onset sepsis, depending on the presentation age and presumed infection mode [10] (Table 1). If signals of infection occur before 3 days of life, it is considered early-onset sepsis (EOS) and if it appears following that, and up to 28 days, it is considered late-onset sepsis (LOS) [11,12,13,14]. However, no time point clearly distinguishes EOS from LOS, with some clinicians defending different segregation time points [15].

While EOS is acquired vertically, with neonates becoming infected either in utero or during birth associated with passage through the vaginal canal, LOS is usually related to the postnatal nosocomial or community environments [21]. Although a reduction of EOS has been observed over the years, probably due to the use of prophylactic intrapartum antibiotics [22], LOS has shown stable or increased incidence, depending on the neonatal units evaluated [21,23]. As such, LOS is still one of the main causes of mortality among preterm infants and of several important morbidities with short- and long-term detrimental effects on neonatal outcomes [24]. In general, LOS affects 1 in 10 very preterm infants (28 to 32 weeks) with incidences reaching as high as 60% in extremely preterm infants (less than 28 weeks) [25]. Furthermore, due to extended hospital stays and additional diagnosis and treatments required, LOS constitutes an important economic burden, which is even higher when considering the costs related to the clinical support that patients may require due to the lifelong impact of the disease.

Due to its transmission venue, microorganisms causing EOS and LOS often differ. While EOS is mainly caused by bacteria present in the maternal vaginal canal, LOS is more likely caused by microorganisms present in neonatal rooms or community environments [13]. As such, considering that each hospital and community have a particular epidemiology profile, microorganisms causing LOS diverge depending on the hospital, country and also over time [21]. In high-income countries, the most frequent causative agents of LOS, especially in extremely and very low birth weight infants, are coagulase-negative staphylococci (CoNS) [21,26,27,28,29,30], accounting for 53 to 78% of the LOS cases [21]. In low- and middle-income countries, although CoNS are still responsible for about 35 to 47% of LOS, *Staphylococcus aureus*, *Klebsiella pneumoniae* and *Escherichia coli* show higher incidence [31,32,33]. However, the lack of studies in low- and middle-income countries makes accurate estimations difficult.

CoNS are a group of staphylococcal species that naturally inhabit healthy human skin and mucosa [34,35,36]. While the CoNS group is composed of more than 50 species [37], *Staphylococcus epidermidis, Staphylococcus haemolyticus* and *Staphylococcus capitis* are the most common etiological agents of LOS [38,39,40,41,42,43,44,45]. The straight relationship between CoNS and the emergence of LOS has to do, in part, with these bacteria’s ability to attach to the surface of medical devices and form biofilms, which is considered the major virulence factor of this group of bacteria [46,47,48,49]. One of the major concerns related to CoNS infections is the increasing resistance to antibiotics observed among this bacterial group, which is further aggravated not only by the phenomenon of multidrug resistance [50,51] but also because CoNS biofilm cells are inherently more tolerant to antibiotics than free-living cells [52,53]. Consequently, the treatment of CoNS infections is becoming increasingly challenging.

Given the significant social and economic implications of LOS, the development of both prophylactic and therapeutic measures has continually been explored; however, so far, antibiotics remain the only form of treatment. Considering that the number of preterm infants is increasing worldwide [1] and with the rapid spread of antimicrobial resistance [54], the management of CoNS infections is likely to deteriorate. Therefore, this narrative review evaluates the current challenges in diagnosis and emerging therapies for the treatment of LOS, in particular the ones caused by CoNS biofilms.

## 2. CoNS Role as Commensal in Newborns

CoNS are normal inhabitants of healthy human skin and mucosa of the respiratory tract and intestine, being one of the most abundant colonizers of all skin sites [34,55]. CoNS are involved in the maintenance of skin integrity and homeostasis, preventing colonization by opportunistic pathogens [56,57,58]. However, the immune system of newborns presents significant differences from that of adults [59,60], such as functional and quantitative deficiencies in antigen-presenting cells and phagocytes, lack of antigenic exposure, less production of pro-inflammatory cytokines and tissue-protective immunosuppressive mechanisms [61,62,63], and therefore the protective role of CoNS colonization is unclear. It could be expected that after contact with microorganisms, the neonates’ host immune system would mount an inflammatory response. However, although it is important to fight the infection, it is also essential to enable the establishment of a commensal bacterial community [64]. Hence, newborns present a particular immune response that relates to the unique environmental pressures and challenges of the first days of life [65,66]. This type of response is called disease tolerance, in which there is a reduction of the potential harm to the host without reducing the pathogen burden, a critical balance that enables fighting infections in early life but, at the same time, permits building a commensal community [64,67]. This is supported by the fact that regulatory T cells, which suppress other cells in the immune system to regulate the inflammatory response [68], induce tolerance to *S. epidermidis* colonization during the neonatal period, but not in adults [69]. Furthermore, the fact that neonates can survive significantly higher bacterial loads than adults during active infection [64] is another indicator of the disease tolerance process. Hence, the establishment of a healthy skin microbiome during this early period is essential to avoid colonization by virulent microbes and, thus, contributes to cutaneous homeostasis and the shaping of inflammatory responses [70].

It was shown that upon birth newborns are readily colonized [71], with staphylococcal species having a major representation [70]. However, the skin microbiome of newborns is very dynamic and the early colonization with staphylococci declines over time [70]. Indeed, the skin microbiome of neonates is not fully established, evolving during the first year of life or even beyond [70]. The colonization of neonates in the first hours/days is most likely the result of contact with medical staff, equipment and their parents [72]. The composition of the newborn microbiome is determined by the previous administration of antibiotics, by modes of feeding and delivery [71,73,74,75] and, very likely, depends on geographical and ethnic factors [63]. In addition, it has been suggested that the microbiome is also modulated by the microorganisms that colonize the neonatal intensive care unit (NICU) rooms if the infants are hospitalized for a long time [76,77]. In this context, it was noticed that these preterm infants harbor a higher prevalence of nosocomial isolates and multi-drug resistant strains compared to term infants [74,75].

Dominguez-Bello and collaborators showed that the skin microbiome of infants delivered by Caesarean section relates to that of the mother’s skin and the ones born through vaginal delivery relate to the mother’s vagina [71]. Furthermore, *S. epidermidis* was found to be the predominant bacterium in colostrum and milk from healthy women [78,79,80,81], enabling the passage of the bacterium through breastfeeding [82]. Hence, it was not surprising to find *S. epidermidis* in the first meconium obtained from both term and preterm breastfed neonates [83,84]. Interestingly, it was demonstrated that maternal milk mostly contains *S. epidermidis* isolates negative for the methicillin resistance gene *mecA* [81,85], which may limit the intestinal spread of *mecA*-positive *S. epidermidis* NICU-associated strains that are a major cause of LOS [86]. Hence, as in adults, CoNS seem to play an important role in the defense of neonates by competing with more virulent strains/species.

## 3. CoNS Role as a Pathogen in Newborns

Although LOS caused by CoNS seems to present less neonatal morbidity rates than LOS caused by other Gram-positive or Gram-negative bacteria [24], it is commonly associated with important morbidities such as bronchopulmonary dysplasia, white matter injury, necrotizing enterocolitis and retinopathy of prematurity [3,87,88,89,90,91,92]. In extremely low birth weight infants (<1000 g), an increased risk of cerebral palsy, mental and psychomotor developmental index and growth impairment was observed [24,87]. As such, even though CoNS can have a benign relationship with the host, the pathogenic lifestyle of CoNS needs to be further explored.

### 3.1. Risk Factors for LOS Caused by CoNS

**Figure 1:**
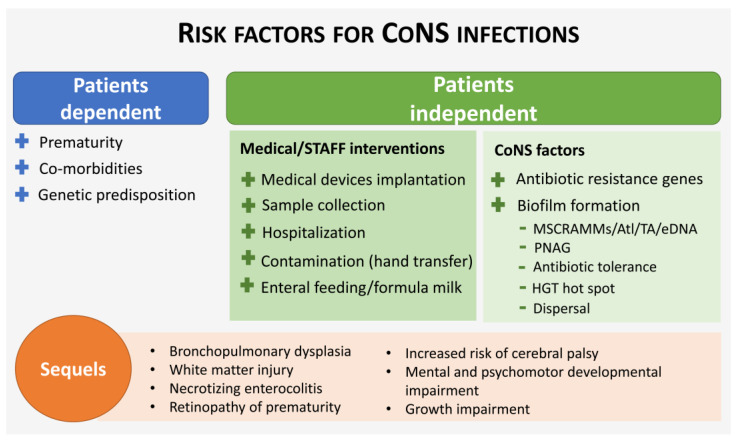
Summary of the several aspects contributing to the development of CoNS-caused infections in preterm infants. MSCRAMMS, microbial surface components recognizing matrix molecules; Atl, autolysins; TA, teichoic acids; eDNA, extracellular DNA; PNAG, poly-N-acetylglucosamine; HTG, horizontal gene transfer.

Preterm infants are more likely to develop a generalized life-threatening infection due to their theoretically immunocompromised condition, in which several components of the immune system are not fully developed at birth [63]. However, the high frequency of LOS caused by CoNS in preterm infants is also related to the fact that they are more likely to require prolonged use of central catheters, parenteral nutrition and mechanical ventilation, which are increased risk factors for CoNS infection [14,25,93,94] (Figure 1). In that sense, it was found that the analyses of bodily fluids such as blood, which are often necessary for diagnosis and implicate the use of invasive procedures, can increase the risk for CoNS-related LOS independently [95]. Recently, it was demonstrated that umbilical catheters are another vector for skin microflora transmission of CoNS into the bloodstream, via biofilm formation [96].

While the use of indwelling medical devices generates an increased risk for CoNS-related LOS, one-third of LOS cases are not associated with medical devices [97], suggesting other risk factors/sources of infection. For instance, the use of formula feeding [95,98,99] and parenteral nutrition with glucose can increase the risk of preterm infants being infected with CoNS [100]. Gut colonization is another route of contamination in preterm infants with immature intestinal mucosal barriers and dysbiosis [101,102]. The gut of preterm neonates becomes colonized, within the first days, with *S. epidermidis* and *S. haemolyticus* carrying the *mecA* gene, which is associated with an increased risk of infection [103]. These strains spread in the NICU afterwards causing LOS [102,104,105]. Indeed, it was shown that *S. epidermidis* can translocate from the gastrointestinal tract into the bloodstream causing infection [101,102]. Furthermore, it was found that *S. capitis* also colonizes the gut of neonates. Butin et al. observed that among the neonates evaluated, 36% had at least one stool culture positive for *S. capitis* during hospitalization [106]. However, differently than *S. epidermidis* or *S. haemolyticus*, the presence of *S. capitis* in the gut microbiota was not a risk factor for LOS development. This suggests that the gut may serve as a reservoir of *S. capitis* but this is not enough to induce LOS [106]. The presence of such strains/species in the gut before the emergence of LOS [102,104,105,107] indicates that alterations in the gut microbiota could prevent the development of LOS [86]. As detailed in Section 2, this could be achieved by feeding neonates with maternal milk that is rich in CoNS that are *mecA* negative [80,85].

Finally, the colonisation rate among gynecological surgical staff with methicillin-resistant CoNS constitutes another important route of contamination of preterm babies. Indeed, it has been shown that the hands of healthcare personnel are the major sources of *S. epidermidis* transmission among neonates [97,102,108]. Thus, hygiene policies need to be revisited and strictly followed to decrease this venue of contamination.

### 3.2. CoNS Virulence Factors

The virulence potential of bacteria is normally defined by their capacity to produce toxins. In the case of CoNS species, although they produce and secrete toxins and exoenzymes, they normally rely on immune evasion mechanisms to cause infection [109]. One of the major factors contributing to CoNS pathogenesis is their ability to form biofilms [37,47,48], as biofilm-related infections are often chronic, persistent and, therefore, difficult to treat. As such, toxins, exoenzymes and all the molecules involved in biofilm formation are often considered good targets for the development of prophylactic and/or therapeutic strategies against CoNS infections.

#### 3.2.1. Toxins and Exoenzymes

Toxin production in CoNS is mainly restricted to the production of phenol-soluble modulins (PSMs). PSM are peptides with surfactant properties that are produced by all Staphylococcal species; however, every species produces a specific repertoire, with some but not all sharing sequence similarities [110]. Studies in *S. epidermidis* and *S. haemolyticus* have shown that PSMs have multiple functions, including in pathogenesis by inducing neutrophils chemotaxis, cytokine release and leukocytes and erythrocytes lysis [111,112,113]. Importantly, Qin et al. have demonstrated, for the first time, that sepsis due to *S. epidermidis* resistant to methicillin is mediated, to a large extent, by the PSM-mec peptide toxin [114]. Of note, since PSM-mec is encoded by *psm-mec* gene locus located in the mobile genetic element staphylococcal chromosome cassette *mec* (SCCmec), which carries the *mecA* gene, the PSM-mec occurs in a series of methicillin-resistant CoNS [115], which are widespread in the clinical setting, specifically in the NICU [97,102,104,108].

Also contributing to CoNS pathogenicity is the ability to produce and secrete exoenzymes. In *S. epidermidis* the metalloprotease SepA plays an important role in the protection against the host immune system response, in particular against antimicrobial peptides (AMPs) and neutrophils [111,116]. Moreover, *S. epidermidis* produces an endopeptidase, Esp, that can degrade complement factors [117] and lipases (GehC and GehD) that facilitate bacteria survival in host fatty acid secretions [118,119,120]. In addition, it was shown that *S. epidermidis* produces a fatty acid-modifying enzyme that inactivates host fatty acids harmful to bacteria [121].

#### 3.2.2. CoNS Biofilm Formation

Biofilms are recognized as the common form of microbial growth [122], including during infection (source of ~65% of medical device-related nosocomial infections [123]), conferring protection against the host immune system [124] and antibiotics challenges [125]. However, the role of biofilm formation by CoNS in the outcome of LOS is unclear, mainly because of the difficulty, both practical and ethical, of studying newborns, especially preterm. Yet, it is known that biofilms are formed right after the implantation of medical devices and that the persistence of biofilms may provide a basis for a continuous release of bacteria into the bloodstream [126,127]. Hence, it is likely that biofilms formed on medical devices are the source of persistent LOS [92]. 

**Figure 2:**
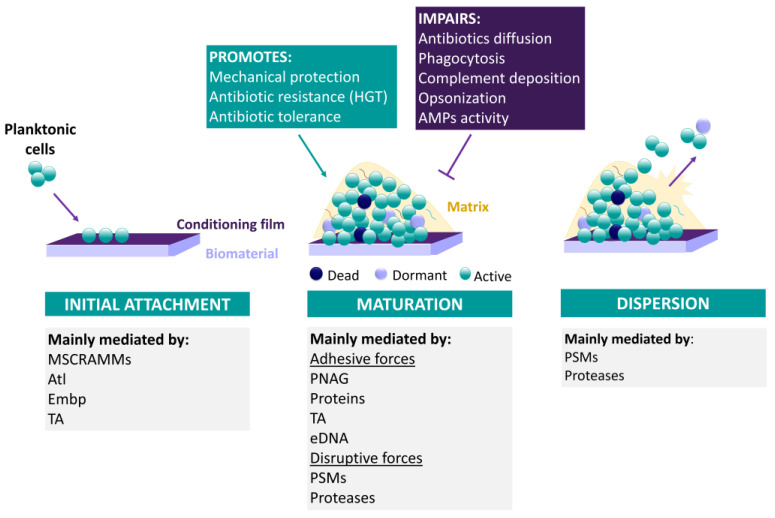
Schematics representing CoNS biofilm formation main steps and molecules involved. HTG, horizontal gene transfer; AMPs, antimicrobial peptides; MSCRAMMS, microbial surface components recognizing matrix molecules; Atl, autolysins; Embp, extracellular matrix binding protein; TA, teichoic acids; PNAG, poly-N-acetylglucosamine; eDNA, extracellular DNA; PSMs, phenol-soluble modulins.

Biofilms are defined as complex communities of bacteria surrounded by a protective matrix, attached or not to a surface [128]. Biofilms are formed following three main stages; (i) initial attachment, (ii) maturation and (iii) dispersal [129] (Figure 2). As can be expected, the first stage is only important in the case of biofilms attached to a surface, such as medical devices, which is the focus of this review. Although CoNS can be attached directly to the medical device surface, in vivo, medical devices are readily coated by host proteins. As such, CoNS normally form biofilms through direct interaction with host matrix proteins [130]. This process is mostly mediated by MSCRAMMs, i.e., microbial surface components recognizing adhesive matrix molecules [131,132], such as serine-aspartate repeat protein G (SdrG) and F (SdrF) [133]. In addition, Atl-type autolysins [134], extracellular matrix binding protein (Embp) [135] and wall teichoic acids [136] also aid the adhesion of *S. epidermidis* to host matrix proteins or tissue, respectively.

Having successfully attached to the surface, bacteria start to proliferate and secrete molecules that will give rise to the protective matrix, entering the second stage of biofilm formation, biofilm maturation [137]. The matrix is composed of several molecules including proteins, polysaccharides, teichoic acids, as well as DNA originating from lysed cells. Depending on the composition of the matrix, biofilms can be categorized as either polysaccharide- or protein-dependent biofilms. Importantly, the composition of the matrix can be modulated by environmental factors such as substrate availability and shear forces [137]. It was observed that *S. epidermidis* isolates recovered from environments with high shear forces are mainly polysaccharide-dependent in contrast with the ones isolated from low-shear environments [138]. In CoNS biofilms, notably in *S. epidermidis*, the polysaccharide poly-N-acetylglucosamine (PNAG), is one of the most prominent molecules [139] and up to 60% of the clinical isolates are known to produce PNAG [140,141,142,143,144]. PNAG, synthesized by the proteins encoded in the genes *icaADBC* [145], has a positive net charge, enabling the interaction with negatively charged molecules, thereby creating the distinguishing sticky characteristic of the matrix.

Although *S. epidermidis* biofilm formation mainly relies on PNAG, as referred to above, biofilms can also be formed through the mediation of proteins such as accumulation–associated protein (Aap) [146], biofilm homologue protein (Bhp) [147] and Embp [135]; however, these biofilms are less robust. Importantly, in addition to the adhesive molecules that maintain the biofilm structure, it is also necessary to generate channels for water and nutrients to reach the deeper layers of the biofilm, as well as to eliminate waste. These channels are shaped by the action of proteases (SepA, Esp, EcpA) [117,148,149,150], nucleases and molecules that disrupt non-covalent interactions, notably PSMs [127,151]. Depending on the extension of the action of these molecules, biofilm structuring can lead to the third stage of the biofilm lifecycle, biofilm dispersal. This last cycle is important to maintain the biofilm lifecycle, as the cells can colonize new locations and establish other foci of infection. 

##### Biofilm Formation as an Antibiotic Tolerance/Resistance Mechanism

As recently defined by the consensus statement on the definitions and guidelines for research on antibiotic persistence, while antibiotic-resistant cells are defined as cells that can grow in the presence of antibiotics by carrying a resistance factor, antibiotic-tolerant cells are defined as cells that can survive antibiotic treatment (slower killing), without carrying a resistance factor, and can regrow after removal of the antibiotic; these tolerance factors can be either environmental or genetic [152]. At the beginning of biofilm studies, it was shown that biofilm cells were 10 to 1000 times more tolerant to antibiotics than their planktonic counterparts [153]. From then on, several studies were performed to identify the mechanisms allowing such a level of tolerance. Among them is the metabolic heterogeneity within biofilm cells [154,155]. Indeed, Rani and collaborators have characterized the spatial distribution of DNA replication, protein synthesis and oxygen concentration on staphylococcal biofilms and four subpopulations of cells were identified: (i) aerobically growing, (ii) fermentatively growing, (iii) dormant and (iv) dead cells, which accounted for ~10% of the total number of cells [156]. This heterogeneity is associated with the particular chemical and nutrient gradient within the several layers of the biofilm, resulting in different gene expression patterns and, thus, cells in different metabolic states [157]. Such a heterogeneous pattern of growth within a biofilm contributes to the alteration of the susceptibility profile to antibiotics. Indeed, this heterogeneity promotes the emergence of cell subpopulations with physiological characteristics which render them resilient to certain antibiotics [154,155]. As such, antibiotics whose targets are actively growing cells, i.e., actively dividing and synthesizing nucleic acids and proteins, will fail to act on slow-growing cells and/or cells with low metabolism [158]. 

Another important mechanism that hinders the activity/efficiency of antibiotics is the matrix. While it was demonstrated that many antibiotics such as vancomycin and cefotaxime have impaired capacity to penetrate the matrix of Staphylococcal biofilms [159,160], the matrix can also function as a decoy, trapping antibiotics [161,162]. In addition, due to the negative charge of polysaccharides and extracellular DNA, the matrix may interfere with the ability of positively charged antibiotics to act [163]. The matrix may also function as a reservoir of enzymes with activity against antibiotics, such as beta-lactamase [164] and aminoglycoside-modifying enzymes [162]. Interestingly, these enzymes are mainly accumulated in the outer layers of the biofilm, constituting the biofilm’s first line of defense [165]. Furthermore, PNAG-positive *S. epidermidis* strains showed increased resistance to several antibiotics and the cell wall degrading enzyme lysostaphin, as compared to PNAG-negative strains [166,167,168]. Noteworthy, sub-inhibitory concentrations of some antibiotics resulted in increased transcription of the *ica* locus presumably leading to higher contents of PNAG [169].

Concerning antibiotic resistance in biofilms, horizontal gene transfer (HGT) is one of the most important mechanisms of exogenous DNA acquisition in staphylococci and, thus, of obtaining antibiotic resistance genes [170]. Biofilms are major mediators of HGT due to the (i) high number and (ii) proximity of cells, (iii) high genetic competence and (iv) greater availability of exogenous DNA [171]. Indeed, it has been reported that the horizontal transfer of resistance-conferring genes is 700 times more efficient in biofilm than in free-living cells [172], further exacerbating the spread of antibiotic resistance [123]. Furthermore, mutations play an important role in the development of antibiotic resistance in staphylococcal species [173] and it was shown that biofilm cultures present increased mutation frequencies [174]. Ryder et al. have shown that *S. epidermidis* biofilm cells presented 4 times higher mutability than planktonic cells, increasing in this way their resistance to rifampicin [174].

##### Biofilm Formation as an Immune System Resistance Mechanism

In addition to the protection against antibiotics, biofilm formation has also an important role in the defense of biofilm cells from the host immune system attack [175]. Biofilm cells are known to present a decreased ability to stimulate the innate immune system, resulting in a lower inflammatory response. This response is mediated by several mechanisms that include the presence of dormant cells [176,177], limited expression of toxins with proinflammatory effects (namely PSMs) [151,178] and the presence of PNAG [175]. In the case of *S. epidermidis*, it was shown that biofilms with higher proportions of dormant cells presented a decreased ability to induce the production of pro-inflammatory cytokines by in vitro cultured bone marrow-derived dendritic cells [176] and murine macrophages, either in vitro or in vivo [177]. Interestingly, it was found that *S. epidermidis* isolates obtained from device-related infections, mainly joint infections [178], often present a mutation in the quorum sensing Agr system that renders it dysfunctional. As such, since PSMs are under strict regulation of the Agr system [179], they are not produced; this has consequences not only for CoNS pathogenicity but also in biofilm structuring, presenting higher biofilm formation in vivo [178]. Hence, it seems that disabling the Agr system enhances the success of the bacterium in vivo. Furthermore, a recent study has followed the evolution of an *S. epidermidis* strain over the 16 weeks of pacemaker-associated endocarditis, and the capacity to form biofilm increased over time, as determined by in vitro assays [180]. Moreover, several mutations were detected, including in the Agr system [180]. Regarding PNAG, besides its important function in biofilm formation and maintenance, it also has a fundamental role in immune evasion. PNAG was shown to protect *S. epidermidis* biofilm cells, in in vitro assays, from neutrophil and AMPs killing [181], antibody-mediated phagocytosis [182] and reduced deposition of complement proteins and IgG on the bacterial surface [183]. In addition, PNAG was shown to present a protective role in vivo, using animal models [183,184,185,186]. Furthermore, ex vivo experiments using cord blood samples reported that the levels of interleukin-6 (IL-6) in blood cultures incubated with a PNAG-positive *S. epidermidis* strain were lower as compared to that of a PNAG-negative strain [187]. Similar results were observed in the blood of adults [188].

## 4. LOS Diagnosis and Treatment

All the mechanisms described above contribute to the development of CoNS biofilm-associated infections, with a chronic and persistent nature, which are very hard to cure with current antibiotic therapy. Furthermore, due to the commensal nature of CoNS [189] and the characteristic metabolic heterogeneity of the cells within biofilms [37,190], the infections caused by this group of bacteria are difficult to diagnose, increasing their economical and clinical burden. As such, it is important to comprehend the methods available for the diagnosis and treatment of CoNS biofilm-based infections, to be able to identify the limitations and, subsequently, develop efficient strategies to detect and treat CoNS-related infections, such as LOS.

### 4.1. Diagnosis of LOS

**Figure 3:**
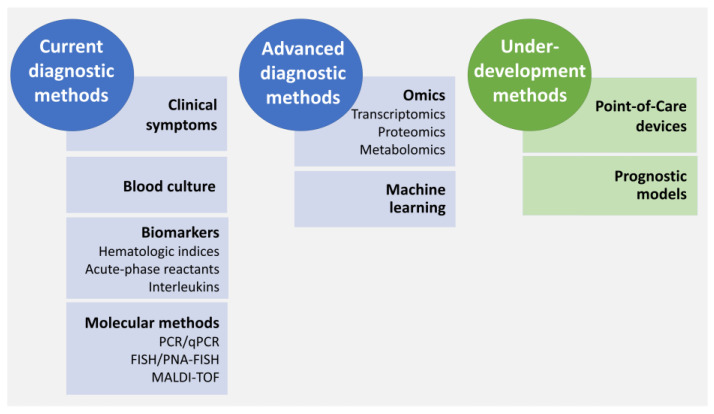
Summary of some of the currently used methods used for diagnosis of LOS, along with advanced and under-development methods. Of note, the list of techniques detailed under “current diagnostic methods” includes methods that are normally used alone, such as blood culture and those that are used as “add-on” tests, such as biomarkers.

Despite the fair advances made in medicine over the years, LOS is still challenging to quickly and accurately diagnose, leading to delays in the application of an adequate treatment, which in turn may result in a higher risk of complications and, ultimately, a higher mortality rate [191]. This difficulty is attributed to several factors such as (i) variable or non-specific symptoms displayed by neonates [21,192], (ii) the maternal intrapartum antibiotic therapy that can mask the presence of bacteria [193] and (iii) the technical limitations of the diagnosis methods currently used [26,194]. Currently, the gold standard for the diagnosis of LOS is the identification of the causative agent through culture methods, namely from blood samples [195,196]. However, this method has time limitations resulting in a delayed diagnosis. As such, to try to speed up the diagnosis, other indicative analyses such as the use of molecular methods to help identify the causative agents, quantification of inflammatory molecules and the evaluation of clinical symptoms are often combined. Nevertheless, a timely and unambiguous diagnosis of LOS is still not available and, thus, there are still attempts to find new diagnostic strategies [12]. Figure 3 briefly summarizes the current, advanced and under-development diagnostic methods for the diagnosis of LOS.

#### 4.1.1. Clinical Symptoms

The symptoms or clinical signs of NS, including LOS, are very comprehensive and can include symptoms that can naturally occur in preterm neonates [197]. In general, symptoms include, among others: (i) temperature instability, (ii) feeding difficulties/intolerance, (iii) apnoea and tachypnea, (iv) respiratory rate or desaturations, (v) heart rate variability, (vi) hypotension, (vii) irritability or seizures [15,20,192,198,199,200]. However, because of the variable or non-specific symptoms displayed by neonates [21,192], further analyses are necessary to be able to accurately diagnose LOS. However, the diagnosis of LOS can be made solely based on clinical symptoms, which is designated as clinical sepsis [195,196]. 

#### 4.1.2. Biological Samples Culture

LOS is confirmed by isolating the causative agent from sterile body sites such as blood, urine and cerebrospinal, pleural, joint, and peritoneal fluids [15]. Blood cultures are the current gold standard for LOS diagnosis. However, the long turnaround time (up to 72 h or 48 h if automated systems are used), the small volume of blood and the reduced number of samples that can be drawn from preterm neonates, as well as the intermittent bacteremia, render this method often insufficient and insensitive [193]. Since most of the deaths occur within the first three days after blood cultures are obtained, faster and more sensitive identification of the causative pathogen is essential for early diagnosis and faster guidance of therapy [14]. In the case of CoNS biofilm-related infection, there are two additional limitations. On one hand, due to their commensal nature in human skin, CoNS-positive blood cultures are often interpreted as contamination during sample collection. To avoid misdiagnosis, specific criteria to define CoNS sepsis were delineated and although they may vary depending on the country and clinical staff, the most well-accepted criterion to consider LOS caused by CoNS is to obtain two positive blood cultures obtained within 2 days [14,201]. On the other hand, as described before staphylococcal biofilms are characterized by the presence of cells with different metabolic states. These include, among others, viable but non-culturable cells that, as the name indicates, are viable but cannot grow in culture media. Thus, despite being alive, these cells are not detected by culture-based methods hindering the interpretation of culture results [190]. Overall, these limitations have triggered the search for additional diagnosis methods, specifically culture-independent techniques.

#### 4.1.3. Biomarkers—Inflammatory Molecules and Hematologic Indices

Considering that the host immune system reacts to the presence of microorganisms, it is reasonable to expect that the characterization of the molecules produced in the context of infection can support the diagnosis of NS. To be considered good biomarkers, among other characteristics, these molecules need to present high sensitivity (~100%), specificity (>85%) and high positive (>85%) and negative (~100%) predictive values of NS [202,203]. In that sense, over the last years, several studies have investigated the ability of inflammatory molecules to be used as biomarkers such as interleukins (IL-1β, IL-6, IL-8, TNF-α), cell adhesion molecules (CD11b, CD14, CD64), acute-phase reactants (procalcitonin, C-reactive protein, serum amyloid A), as well as hematologic indices such as white blood cells, neutrophils counts and platelets counts [192,202,204,205,206]. Despite the several developments regarding the identification of biomarkers for NS diagnosis, biomarkers specifically for LOS detection are poorly known.

LOS in preterm infants, in particular in extremely preterm infants, is mainly caused by CoNS, which seems to cause less inflammatory response compared with other bacteria [207,208,209]. Consequently, this less virulent profile can impair the application of inflammatory molecules as biomarkers in this case. Recently, Mwesigye and collaborators observed that there is growing evidence that C-reactive protein (CRP), an acute phase reactant synthesized by the liver in response to pro-inflammatory cytokines, is losing strength as a biomarker for LOS [210] despite being the most commonly used and widely implemented biomarker so far [192,204,206,211,212]. Notwithstanding the uncertainties regarding its predictive significance, CRP is still the object of analysis in several studies and it was shown that although CRP alone does not seem to be the best predictive biomarker, in combination with complete blood count, for instance, it has a good sensitivity [213]. However, other studies have shown that serum amyloid A, the expression of CD64 on neutrophils surface, presepsin and endocan have higher potential for a more accurate diagnosis of LOS [210,211,214]. Procalcitonin (PCT), another acute phase reactant, holds promising sensitivity for the prediction of LOS alone [215,216] and in combination with IL-6 [212]. Sherbiny and collaborators have also shown that hepcidin, which controls the levels of systemic iron [217], constitutes another possibility for rapid and accurate diagnostics of LOS [218,219]. Finally, it has been demonstrated that the levels of IL-6, PCT and CRP in CoNS-positive blood cultures were associated with increased mortality and, thus, a combination of these three molecules can establish a good biomarker [207]. Yet, these results were obtained with a small sample, requiring further investigation. Concerning hematologic indices, the immature-to-total and immature-to-mature neutrophil ratios and platelet counts were found to be highly predictive laboratory signs, but immature-to-total seems to be the most reliable [220,221,222]. It is important to stress that the selection of the best biomarker for NS diagnosis, namely LOS, depends on several aspects such as gestational age, time of infection and sampling [223]. Thus, the best biomarkers will depend on the particular clinical situation.

Although advantageous in some respects, the use of inflammatory biomarkers has a downside; the inflammatory molecules detected can be of non-infectious origin confusing the results. As such, these biochemical analyses do not have sufficient diagnostic accuracy to support a decision without considering the results of microbial cultures [224] but constitute a good “add-on” method. 

#### 4.1.4. Molecular Biology Methods—Nucleic Acids Analysis

To try to increase sensitivity and accelerate the diagnosis of LOS, molecular methods based on the detection of nucleic acids (DNA or RNA) are being increasingly used. These are either based on amplification (PCR, qPCR), probes hybridization (fluorescence in situ hybridization) or sequencing techniques (whole generation sequencing) [204]. The simplification of nucleic acids amplification procedures and instruments and the development of automated systems have fostered the use of amplification-based methods in the clinical setting. Therefore, it is not surprising that most of the methods used in neonatal studies are based on the amplification of nucleic acids, followed by hybridization- and sequencing-based methods, which are normally more time-consuming and expensive, respectively. 

Several of the routine molecular methods currently used for LOS diagnosis include kits such as GeneXpert, FilmArray, Verigene, PNA-FISH and QuickFISH, which can yield results in as little as 3 h [225]. Although these kits enable the detection of CoNS, due to the relevance of CoNS in the development of LOS, there are kits specifically developed to detect *Staphylococcus epidermidis*, *Staphylococcus hominis*, *Staphylococcus haemolyticus*, *Staphylococcus saprophyticus* and *Staphylococcus capitis*, which are among the most frequently isolated in the skin microbiome and important in the context of LOS [34,48,49]. However, these methods rely on positive blood cultures and, thus, although fast, these methods do not constitute a major advantage as they do not preclude the need for previous cultures. In that sense, recent advances in molecular methods have enabled the amplification of nucleic acids directly from biological samples [192,225,226]. Two examples are the kits SeptiFast, from Roche, and SepsiTest, from Molzym, which require 1 to 1.5 mL whole blood to be able to detect, respectively, 25 to more than 300 pathogens at the same time, with a turnaround time of 6 h and 10 h [227]. In the case of SeptiFast, 100 µL of blood can be used but the sensitivity drops to 80% [228].

On the other hand, PCR/qPCR-based methods may result in false-positive results [229] and the reported divergences between blood culture and PCR/qPCR results [230,231], have raised questions about the efficiency of molecular methods. In addition, the sensitivity of these methods depends on the quality of the template (nucleic acids quantity, integrity and purity), which in turn depends on the principle of the methods used for nucleic acids isolation and the quality of the initial samples [229,232]. Furthermore, the quality of the molecular assay is also affected by contamination, either through other organisms or within the laboratory environment, and in situations of low-level bacteremia [233]. Thus, for now, molecular methods have promising potential as complementary tests but not alone [204,224]. 

#### 4.1.5. Molecular Methods—Proteins Analysis

One of the ultimate techniques for bacterial pathogen identification is matrix-assisted laser desorption-ionization/time-of-flight (MALDI-TOF). Although in the past this method required isolated colonies [234], following several developments it can be performed directly from blood, urine, and cerebrospinal fluid samples, significantly improving the turnaround time [235,236,237,238]. Nevertheless, for the direct analysis of clinical samples, additional pre-treatment protocols are necessary to selectively recover bacterial cells [239] since multiple proteins from the host (hemoglobin in blood cultures, for instance) may be present in high quantities [238]. Hence, despite the clear advances, further studies are required to address the current limitations in the detection of microorganisms directly from biological samples. Another promising addition to this method is the ability to analyze the pathogens’ antimicrobial susceptibility, giving results at least 24 h earlier than when identification and/or resistance detection is performed using conventional methods [240]. This feature can greatly improve the treatment of LOS and the outcome of infection [241].

The downside of this method is the initial investment, but the cost of sample processing is cheaper than several of the methods used, mainly when considering the higher number of samples that need to be processed daily in the hospital environment. However, although this is true for high-income countries, it is difficult to implement in developing countries. Moreover, for now, it seems that MALDI-TOF has higher sensitivity to detect Gram-negative bacteria from culture bottles, particularly Enterobacteriaceae, than Gram-positive bacteria [242,243]. As such, improvements are still necessary to accurately detect LOS caused by CoNS and to fully implement this methodology in clinical laboratories.

#### 4.1.6. Diagnosis Approaches under Development

Despite the several methods currently available, there is a continuous search to advance the methods available or for the development of new methods that provide prognostic information or definitive diagnostics promptly. Point-of-Care devices, which can be done at the bedside and can yield rapid results are the Holy Grail of NS diagnosis. The detection of volatile organic compounds in the breath of patients has been shown to have the capacity to differentiate sepsis cases from inflammation [244,245]. Although promising, validation in human studies is necessary [244,245]. Another solution that has been explored is the development of devices that can detect a variety of biomarkers and, this way, obtain a more accurate diagnosis of NS [246]. 

As in any other field of science, Omics technologies and machine learning may be very useful in the advance of the methods used to diagnose NS, including LOS. Transcriptomics [247,248,249,250], proteomics [251,252,253,254,255] and metabolomics [252,256,257,258] have been used to characterize the response of preterm neonates to NS [259,260] and, together with machine learning approaches, can help to identify promising biomarkers, to determine NS risk, treatment response and prognosis [261,262]. Some steps were already made in that direction and Pediatric Sepsis Biomarker Risk Model, PERSEVERE, was developed and validated as a prognostic enrichment tool for pediatric septic shock [260,263]. Ongoing research is evaluating the use of PERSEVERE in the prediction of NS [204].

### 4.2. LOS Treatment

**Figure 4:**
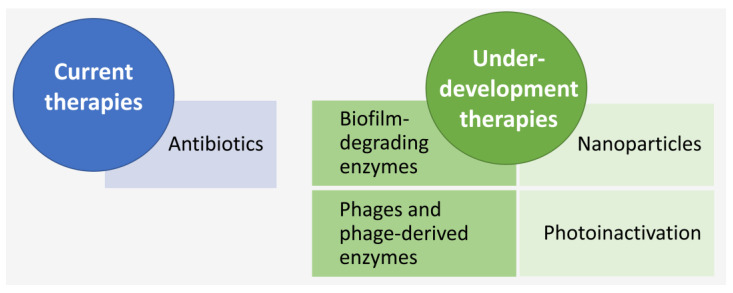
Summary of the current and under-development therapeutic methods for CoNS-associated infections, including LOS. The use of nanoparticles, as well as photoinactivation, which involves the use of visible light, a photosensitizer and oxygen to produce reactive oxygen species and free radicals that create oxidative stress in bacteria [158], have shown promising results in staphylococci biofilms, including methicillin-resistant *S. aureus* [268,269,270,276], but more studies are necessary to investigate their consequences on host cells.

So far, the only available treatment option for NS, including LOS, is the use of antibiotics. As referred to above, due to the lack of methods that can accurately and quickly diagnose LOS, in the case of suspicion of LOS, clinicians initiate empirical broad-spectrum antibiotic treatments [21]. Indeed, several studies covering different countries have reported that most of the treatments for NS do not have a confirmed diagnosis. This can increase antibiotic resistance and, even though it may save lives, it may also result in important sequels for newborns such as reduced gut microbial diversity [264], invasive fungal infections, necrotising enterocolitis [265], inflammatory diseases [266] and increased likelihood of early childhood obesity [267]. Hence, several other therapeutic options, mainly antibiotic-independent, but also including the improvement of the activity and delivery of current antibiotics, with fewer side effects, are being explored. Herein, some of the strategies that are being investigated in the context of CoNS biofilms are outlined (Figure 4), but a more exhaustive list of strategies can be seen in [158,268,269,270]. Of note, due to biofilm characteristic heterogeneity (cells with different metabolic states and under different pH and levels of oxygen and nutrients), the use of combined strategies may increase our likelihood of effectively targeting biofilm infections. As such, there are some developments in the area already, with the combination of antibiotics with other new methods being one of the most common strategies [271,272,273,274,275].

#### 4.2.1. Currently Available Therapies—Antibiotics

As discussed above, in the presence of symptoms and signs suggestive of NS, empiric therapy is initiated [20]. Despite the serious consequences that this approach may hold, it has been proven to reduce both the mortality and morbidity of affected newborns [277]. The most commonly recommended treatment for NS is a β-lactam antibiotic, normally ampicillin, flucloxacillin or penicillin, combined with an aminoglycoside, most frequently gentamicin [20,21]. In the particular case of LOS, a cephalosporin, usually cefotaxime, or a glycopeptide, such as vancomycin, may be used instead due to the increased resistance demonstrated by CoNS to the other antibiotics frequently used to treat NS [21,278,279,280,281]. However, it is important to stress that depending on the local antibiotic resistance of the most common pathogens causing LOS, the antibiotic regimen may change [282]. Importantly, the empiric therapy should be altered as soon as diagnosis results are obtained, and an immediate cessation of antibiotics shall be performed if culture-negative results are obtained and the infant shows no subsequent clinical evidence of NS [283]. The increasing resistance to antibiotics, within the hospital and in the community, is seriously compromising our ability to treat LOS, with CoNS being increasingly resistant to a plethora of antibiotics, especially against beta-lactam antibiotics and, more importantly, frequently presenting multi-drug resistance phenotypes (resistant to 3 or more classes of antibiotics) [283,284,285]. Among *S. epidermidis* neonatal isolates, epidemiological studies have reported that ~52.7–85% present a multi-drug resistance profile, with ~92–99% resistant to penicillin and ~70–78% to gentamicin [43,286,287]. In the case of *S. haemolyticus*, the studies performed showed that ~82–87% of the isolates are multi-drug resistant, with 90% of the isolates being resistant to penicillin and ~61–79% to gentamycin [44,287,288]. Studies analyzing *S. capitis* neonatal isolates are mainly focused on the clone NRCS-A, since it has recently emerged as a major pathogen of LOS, having already been isolated in several countries all over the world [25,38,45]. This clone has shown high resistance to penicillin (99%), methicillin (95.6%) and gentamicin (95.1%) [38]. With regards to vancomycin, all the *S. epidermidis* and *S. haemolyticus* neonatal isolates studied so far have shown 100% susceptibility to vancomycin [43,286,289]. However, in the case of *S. capitis* neonatal isolates, in particular, the ones belonging to the NRCS-A clone showed resistance (37.5%) or heteroresistance (62.5%) to vancomycin [38]. As such, considering that vancomycin is one of the suggested antibiotics to treat LOS, it is not surprising that cases of prolonged *S. capitis* LOS have been reported [290,291]. Thus, alternatives to vancomycin have to be considered [292] and daptomycin and linezolid have been suggested [293,294,295,296]. Yet, Butin et al. reported that vancomycin resistance in *S. capitis* NRCS-A was associated with an increase in daptomycin and teicoplanin minimal inhibitory concentrations, but not in linezolid [297]. This indicates that daptomycin may not be the best approach, but highlights linezolid as a possible alternative for the treatment of LOS caused by *S. capitis* [298]. However, the use of linezolid needs to be conservative as resistance to this drug has already been observed in *S. capitis* isolates collected from non-neonate patients [292] and may not take long to upsurge in neonates.

When in biofilms, CoNS are more tolerant to antibiotics. Indeed, Qu et al. have shown that the resistance of CoNS to several antibiotics increases significantly as the biofilm develops [285]. Thus, although the strains may be susceptible in antimicrobials susceptibility tests, which are performed with planktonic cells, the antibiotic in question may not cure the infection caused by CoNS biofilms. One antibiotic that has performed well against biofilms is rifampicin [299]. However, rifampicin quickly develops resistance when used alone and, thus, it should be used as part of a combined therapy [300,301]. It was shown that when added to penicillin or vancomycin, it is effective in the treatment of refractory cases of bacteremia caused by CoNS in infants [302,303,304]. However, the combination of antibiotics needs to be tested since it may have synergistic but also antagonistic effects [305].

Notwithstanding their importance to treat LOS, the use of antibiotics always entails risks. Vancomycin, for instance, is known to cause fever and phlebitis and, in rare cases, nephrotoxicity and ototoxicity in adults [306]. In addition, an increased risk of nephrotoxicity occurs when amino-glycosides are combined with vancomycin [306,307,308]. Concerning the use of linezolid, care should be taken regarding the development of lactic acidosis and acidemia, which should be carefully monitored [309]. Concerning rifampicin, adverse effects such as hepatotoxicity, renal failure, rash, and hematological abnormalities such as thrombocytopenia have been reported in adults [310]. When it comes to infants, although some studies performed did not detect significant adverse effects of the use of rifampicin [311,312], it is suggested that the level of bilirubin should be monitored during therapy. Moreover, more studies are necessary to ensure proper doses and duration of the use of rifampicin in infants. In addition to the specific adverse effects of each antibiotic, extended use of any antibiotics can increase the risk of developing neonatal candidemia [313,314]. Noteworthy, except when detailed, these studies were performed in adults and, thus, the consequences of the use of these antibiotics in preterm neonates remain unclear. 

Hence, due to (i) antibiotic resistance escalation in CoNS, (ii) biofilm inherent tolerance to antibiotics and a hot spot for HTG, (iii) the increased number of preterm infants and (iv) the fact that antibiotics are the only available treatment solution, CoNS biofilm-related infections are likely to upsurge and will be extremely difficult to eradicate. Therefore, new treatment strategies encompassing antibiotic-independent approaches, but also the alteration of already existing antibiotics or the design of new ones, are urgent. 

##### Improved Antibiotic-Based Treatment Approaches

Although studies exploring new synthetic or altered antibiotics, used alone or in combination, for the treatment of CoNS biofilm-related infections are rare, encouraging results have been shown for *S. aureus*, indicating that the same approach can be applied to CoNS species. Antonoplis et al. have developed a synthetic vancomycin, vancomycin-d-octaarginine conjugate (V-r8), which was able to eradicate 97% of biofilm-associated methicillin-resistant *S. aureus* in a murine wound infection model [315]. Furthermore, another study has altered tobramycin in a way that it was able to kill *S. aureus* persister cells 4 to 6 logs more effectively than the original tobramycin [316]. With regard to the combination of classic antibiotics with new molecules, Kim et al. have shown that synthetic retinoid antibiotics CD437 and CD1530 were able to kill methicillin-resistant *S. aureus* cells, including persister cells, and their efficacy was enhanced in the presence of gentamicin [272]. Furthermore, the combination of bithionol and gentamicin reduced the load of *S. aureus* cells in a mouse model of chronic deep-seated methicillin-resistant S. *aureus* infections [271]. Similar results were obtained with 5-methylindole, which together with tobramycin was able to kill *S. aureus* persisters in a skin wound mouse model and showed good activity in vitro against *S. epidermidis* cells when in combination with gentamicin and kanamycin [273]. 

#### 4.2.2. Antibiotic-Independent Treatment Approaches under Development

Although new treatment strategies are being explored, most are still under preclinical tests. Several recent reviews detail the most promising strategies being developed for the treatment of staphylococcal infection [128,137,158]. As such, herein, only a brief outline of the strategies that can be used in the future to treat CoNS biofilm-related infections is presented. Worth mentioning, the in vivo efficacy of these strategies still needs to be demonstrated.

Among the strategies being explored, there are some strategies that even though they present exciting results in several bacteria, in the case of Staphylococcal species, including CoNS, may not work that well. One of those examples is the use of AMPs. AMPs are very popular due to their strong antimicrobial activity, irrespective of cells’ metabolic state and resistance to antibiotics, and because AMPs are less prone to induce resistance [274]. However, when it comes to the treatment of staphylococcal infections, the use of AMPs may fail as Staphylococcal species have learned to sense AMPs and counteract them efficiently [317,318]. Yet, some studies are reporting the ability of AMPs to treat staphylococcal biofilms, namely *S. aureus* [319]. Hence further studies are necessary to accurately determine the ability of AMPs to treat CoNS-based infections. Another example is the use of molecules with the capacity to block quorum-sensing communications. These molecules may not be the best approach to deal with staphylococcal biofilms, since as was shown earlier the interference with the Agr system may increase biofilm formation [128,178].

##### Biofilm-Degrading Enzymes

Biofilm structure is mainly maintained by the matrix that protects and holds all the cells together. It is also the structure of the biofilm that promotes the different gradients of oxygen and nutrients, responsible for the heterogeneity of the cells within the biofilm. Hence, if this structure is destroyed, it is reasonable to assume that the particularities of the biofilm are lost and, thus, the free cells become more exposed and susceptible. Nevertheless, it is important to consider that the cells detached from the biofilm will likely lead to reinfection in the host [127]. Thus, the use of molecules that lead to biofilm disassembly needs to be used in combination with antibiotics to avoid subsequent regrowth [320,321]. Yet, some studies in *S. epidermidis* have revealed that the cells released from the biofilms are more tolerant to antibiotics and stimulate a more aggressive immune response than their planktonic or even biofilms counterparts [53,322]. Hence, the use of biofilm-degrading enzymes needs to be carefully evaluated. Examples of such biofilm degrading enzymes are dispersin B, produced by *Actinobacillus actinomycetemcomitans*, which degrades PNAG [321,323,324,325]; DNases I, that degrades extracellular DNA; protein K, that degrades proteins [137], and lysostaphin that degrades peptidoglycan from Staphylococcal species, including *S. epidermidis* [326]. In addition, it is important to consider the side effects of applying molecules with broad activity, such as proteases, as these may interfere with host proteins and tissues [327,328] and may have unexpected consequences. 

##### Bacteriophages and Lysins

Bacteriophages (phages) are viruses that infect bacteria and their utilization for the treatment of infections, including biofilm-based, is on the rise due to their safe use in humans and for the environment and because they do not interfere with the healthy microbiota [158,329,330]. This has to do with the fact that bacteriophages (i) present narrow host specificity, preventing the killing of beneficial bacteria, (ii) affect both antibiotic-susceptible and resistant bacteria and (iii) are bactericidal (lytic phages), with their efficacy not relying on biofilm cells’ physiology [331]. Another important feature of phages is the ability to reach and infect the cells within the biofilm. Indeed, González and collaborators have shown that two staphylococcal phages (philPLA-RODI and philPLA-C1C) were able to move across the biofilm and propagate [332]. 

In *S. epidermidis*, the polyvalent Phage K has shown activity against biofilm cells [333] and a more recent study has demonstrated that a novel bacteriophage, DRA88, in combination with Phage K, strongly reduced the biomass of CoNS biofilms [334]. Furthermore, an *S. epidermidis*-specific phage, SEP1, was able to infect scraped biofilms, persister and biofilm-released cells. Interestingly, these findings suggest that its activity was affected by the biofilm matrix [335,336], which may be a downside of phage utilization. However, some staphylococcal phages harbor enzymes with the capacity to degrade polysaccharides, which can surpass the limitation posed by the biofilm matrix [337]. Despite the clear advantages of using phages, attention should be paid to the concentration of phages to be used, as it has been reported that the application of low doses resulted in *Staphylococcus aureus* biofilm development [338,339]. 

In addition to using phages, enzymes encoded by phages, namely lytic proteins such as endolysins and peptidoglycan hydrolases can also be used [158,340]. Similarly to phages, lytic proteins are specific to their target and were able to kill persister cells [341]. For instance, the endolysin LysGH15 was able to disrupt biofilms formed by some LOS-relevant CoNS, namely *S. epidermidis*, *S. haemolyticus* and *S. hominis* [342]. Noteworthy, the efficacy of LysGH15 was evaluated in vivo and a lower bacterial load was detected in both the blood and solid organs of endolysin-treated subjects [342]. CF-301, another endolysin, has also been shown to efficiently disturb biofilms formed by several CoNS species, including biofilms formed by different staphylococcal species [343]. Both phages and lytic proteins may be used alone or in combination with other management methods such as antibiotics. In fact, there are some promising studies in *S. aureus* showing the synergism between phages and antibiotics [275,344,345].

Although not directly related to the natural capabilities of phages to target CoNS biofilm-based infections, phages can be used as tools to deliver other mechanisms capable of interfering with biofilm viability. For instance, the delivery of CRISPR-Cas systems by modified bacteriophages was proposed as a potential strategy in the treatment of staphylococcal infections, including biofilm-based infections [346,347]. However, more studies are necessary to evaluate the CRISPR-Cas system’s impact on the treatment of staphylococcal biofilm infection.

## 5. Conclusions

Notwithstanding being the most frequently isolated Gram-positive bacteria in the context of LOS all over the globe, the infections caused by CoNS are normally perceived as an infection with relatively good outcomes, being often underestimated. However, previous and recent epidemiological studies, as well as the reported short-and long-term neurologic and developmental sequels, have raised awareness of the importance of these commensals as serious neonatal pathogens.

Although a lot of information is available regarding CoNS biofilms, most of these studies were performed in in vitro conditions, and it has become clear that in vitro observations cannot always be transferable to the in vivo level. Furthermore, information regarding CoNS biofilms’ role in NS is very limited. This is mainly related to the lack of in vivo models able to resemble preterm infants’ immune systems. As such, more in vivo studies are necessary to enable full comprehension of the role of CoNS biofilms in LOS outcomes. To surpass the limitations of working with reduced neonatal samples, omics and machine learning analysis can be of help.

Another complication associated with CoNS biofilm infections is the increased resistance to antibiotics that have been seen among these species, including multidrug resistance, together with the higher tolerance of biofilm cells. Despite the several advances made in the development of antibiotic-independent treatment strategies, most still need in vivo validation, followed by preclinical trials, anticipating that new treatment strategies will only be available in the market many years from now.

For all the aforementioned reasons, it is important to emphasize that despite all the studies that can be completed to comprehend the pathogenicity of CoNS biofilm infections and the response of preterm infants in the context of LOS, one of the most important aspects in the reduction of neonatal infection lies in preventive measures [24]. Therefore, the prevention of preterm births, prolonged use of antibiotics, and use of invasive procedures disrupting skin microbiome, as well as promotion of early feeding with breast milk and strict hand hygiene may constitute the best allies for the control of LOS caused by CoNS [24].

## Figures and Tables

**Table 1:** Compilation of the differences that can be found between EOS and LOS [12,16,17,18,19,20].

Characteristics	EOS	LOS
Onset	0–3 days	3–28 days
Transmission	Vertical (female genitourinary system)	Horizontal (neonatal/community environments)
Frequent causative agents	Group B *Streptococcus (GBS)*	CoNS
	*Escherichia coli*	*Staphylococcus aureus*
	*Haemophilus influenza*	*Escherichia coli*
	*Listeria monocytogenes* (dietary intake)	*Klebsiella pneumonia*
		*Acinetobacter baumannii*
Risk factors	- Maternal GBS colonization	- Prematurity
	- Chorioamnionitis	- Low birth weight
	- Delivery before 37 weeks	- Prolonged use of catheters/medical devices
	- Premature rupture of membranes	- Invasive procedures
	- Prolonged rupture of membranes (>18 h)	- Prolonged antibiotic therapy
	- Multiple gestations	
	- Preterm birth (<37 weeks)	
Mortality	3–16%	36–52%

## Data Availability

Not applicable.

## Abbreviations

**Abbreviation**	**Definition**
NS	Neonatal sepsis
EOS	Early-onset sepsis
LOS	Late-onset sepsis
CoNS	Coagulase-negative staphylococci
GBS	Group B Streptococcus
NICU	Neonatal intensive care unit
MSCRAMMs	Microbial surface components recognizing matrix molecules
Atl	Autolysins
TA	Teichoic acids
eDNA	Extracellular DNA
PNAG	Poly-N-acetyl-glucosamine
Embp	Extracellular matrix binding protein
HGT	Horizontal gene transfer
Srd	Serine-aspartate repeat proteins
Aap	Accumulation-associated protein
Bhp	Biofilm-homologue protein
SepA	*S. epidermidis* metalloprotease A
Esp	*S. epidermidis* serine protease
EcpA	*S. epidermidis* protease A
PSM	Phenol-soluble modulins
AMPs	Antimicrobial peptides
CRP	C-reactive protein
PCT	Procalcitonin
PCR	Polymerase chain reaction
qPCR	Quantitative-polymerase chain reaction
FISH	Fluorescence in situ hybridization
PNA FISH	Peptide nucleic acid fluorescence in situ hybridization
MALDI-TOF	Matrix-assisted laser desorption-ionization/time-of-flight
CRISPR-Cas	Clustered regularly interspaced short palindromic repeats
